# Lower extremity amputation protocol: a pilot enhanced recovery pathway for vascular amputees

**DOI:** 10.1016/j.jvscit.2022.08.003

**Published:** 2022-08-18

**Authors:** Leigh Ann O’Banion, Yazen Qumsiyeh, Heather Matheny, Sammy S. Siada, Yueqi Yan, Jade S. Hiramoto, Cambia Rome, Rachel C. Dirks, Anne Prentice

**Affiliations:** a Division of Vascular Surgery, Department of Surgery, University of California, San Francisco-Fresno, Fresno, CA; b Health Science Research Institute, University of California, Merced, Merced, CA; c Division of Vascular Surgery, Department of Surgery, University of California, San Francisco, San Francisco, CA

**Keywords:** Amputation, ERAS, LEAP, Vascular amputee

## Abstract

Vascular patients, an inherently older, frail population, account for >80% of major lower extremity amputations (transtibial or transfemoral) in the United States. Retrospective data have shown that early physical therapy and discharge to an acute rehabilitation facility decreases the postoperative length of stay (LOS) and expedites ambulation. In the present study, we sought to determine whether patients treated with the lower extremity amputation protocol (LEAP) will have improved outcomes. We performed a nonrandomized prospective study of vascular patients undergoing an amputation from January 2019 to February 2020. Patients who were nonambulatory or had undergone a previous contralateral major amputation were excluded. LEAP is a multidisciplinary team approach to the perioperative care of amputees using an outlined protocol. The prospective patients were compared with historic controls treated before the initiation of LEAP (January 2016 to December 2018). The primary outcomes included the postoperative LOS, time to receipt of a prosthesis, and time to ambulation. Of the 141 included patients, 130 were in the retrospective group and 11 in the LEAP group. The demographics and comorbidities were similar. All 11 LEAP patients had undergone a below-the-knee amputation, with 1 requiring revision to an above-the-knee amputation. Of the 130 retrospective patients, 122 (94%) had undergone a below-the-knee amputation, with 1 requiring revision to an above-the-knee amputation. The LEAP patients were more likely to be discharged to acute rehabilitation (100% vs 27%; *P* < .001), receive a prosthesis (100% vs 45%; *P* < .001), and ambulate with the prosthesis (100% vs 43%; *P* < .001). The LEAP patients had received physical therapy 2 days sooner than had the retrospective controls (*P* = .006) with a shorter postoperative LOS (3 days vs 6 days; *P* < .001). Of the patients who had received their prosthesis, the LEAP patients had received their prosthesis, on average, 2 months sooner than had the retrospective cohort (81 ± 39 days vs 137 ± 97 days, respectively; *P* = .002) and had ambulated with their prosthesis sooner (86 ± 53 days vs 146 ± 104 days, respectively; *P* = .002). No differences were found in the incidence of surgical site complications or unplanned readmissions between the two groups. The results from the present pilot study have demonstrated that the use of LEAP can significantly decrease postoperative LOS and expedite the time to independent ambulation with a prosthesis for vascular patients undergoing a major lower extremity amputation. These findings suggest a powerful ability to bridge the healthcare gap for this high-risk, underserved, and ethnically diverse population using a disease-specific standardized protocol.

Patients with vascular disease account for >80% of all lower extremity amputations in the United States.^1^, ^2^, ^3^ In 2019, the estimated cost of a major amputation in the United States was >$89,000/patient. With >1.6 million people living with an amputation, this burden cannot be overstated.^4^, ^5^, ^6^ Also, >50% of these amputations will be major (transtibial or transfemoral), both of which have significant potential for disabling outcomes related to ambulation and independent living. Additionally, patients who require a major amputation have had significantly higher mortality rates than age-adjusted patients from the general population (69% and 34% at 1 and 5 years, respectively).^7^

Patients requiring a major lower extremity amputation because of vascular pathology will generally be older with numerous comorbidities, including uncontrolled diabetes mellitus, advanced coronary artery disease, and renal failure. They tend to be more frail and predisposed to deconditioning compared with young traumatic amputee patients.^3^^,^^8^ The former patients are also less likely to be functionally ambulatory after amputation.^9^ The avoidance of prolonged bed rest is a modifiable risk factor that can improve functionality, decrease the hospital length of stay (LOS), and reduce the overall morbidity and mortality rates in this frail population.^10^ Patients who can safely transfer from a bed to wheelchair have had fewer complications of prolonged immobility, a shorter hospital LOS, and been able begin ambulating with a prosthesis more quickly.^11^, ^12^, ^13^ Retrospective data have also suggested that early physical therapy and discharge to an acute rehabilitation facility can decrease the postoperative hospital LOS and time to ambulation with a prosthesis.^1^

Multidisciplinary teams using a standardized perioperative protocol to improve pain control, increase early mobility, and shorten the LOS have been shown to be critical to improving the long-term outcomes for surgical patients.^11^, ^12^, ^13^ Numerous studies have shown that the use of enhanced recovery after surgery (ERAS) protocols, irrespective of disease pathology, can optimize patient care. These standardized protocols have resulted in faster recovery, fewer readmissions, and increased staff and patient satisfaction.^14^, ^15^, ^16^ Multiple studies have also shown that the use of a specific protocol or pathway after amputation will decrease the overall hospital and patient costs. However, at present, no standardized protocol is available for these specific patients.^13^ Thus, we developed the lower extremity amputation protocol (LEAP) using core ERAS principles to improve and standardize the care for vascular amputees (Fig 1). The purpose of the present study was to evaluate the postoperative outcomes after major lower extremity amputation using a standardized protocol (LEAP).

**Fig 1:**
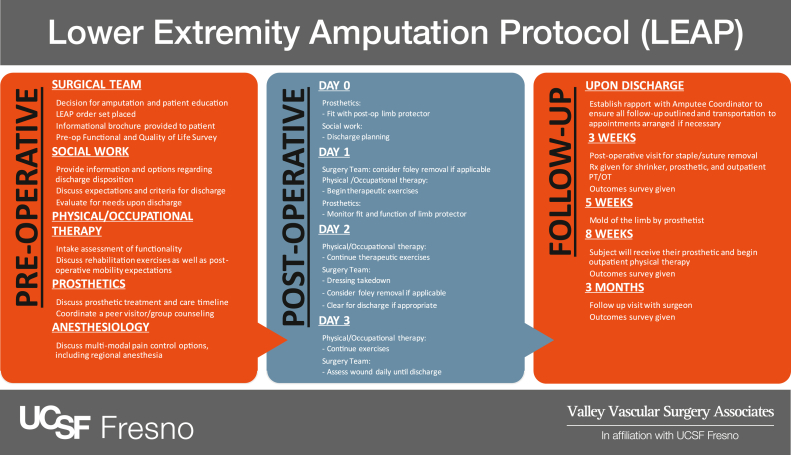
Lower extremity amputation protocol (LEAP) model. *Post-op,* Postoperative; *Pre-op,* preoperative; *PT/OT,* physical therapy/occupational therapy; *Rx,* prescription; *USCF,* University of California, San Francisco.

## Methods

We performed a prospective, nonrandomized pilot trial at the Community Regional Medical Center, a tertiary-level safety net hospital in Fresno, California, from January 2019 to February 2020. The present study included consecutive patients who had presented with clinical WIfI (wound, ischemia, foot infection) stage 5 (unsalvageable foot). The included patients had a known diagnosis of peripheral arterial disease (PAD), defined as an ankle brachial index ≤0.9 or diabetes mellitus with a noncompressible ankle brachial index requiring a major lower extremity amputation, defined as transtibial or transfemoral. Trauma and burn patients, patients who were nonambulatory before surgery, and those who had previously undergone a contralateral major amputation were excluded. No patient was excluded because of insurance status or housing situation. The institutional review board at the Community Regional Medical Center and University of California San Francisco, Fresno, approved the present study. All data points were collected from the electronic health records, and the data were stored and secured in a REDCap database (REDCap, Nashville, TN).

Patients requiring a major lower extremity amputation were enrolled into the prospective arm of the present study if they had received a diagnosis of PAD and provided written informed consent to enrollment. These patients’ treatment course then followed an outlined multidisciplinary protocol, LEAP, implemented in a comprehensive three-tiered approach: preoperative, postoperative, and outpatient follow-up (Fig 1). After patient enrollment, an electronic health record order panel was placed, notifying all the disciplines involved. Preoperatively, the surgical team educated the patients regarding their disease process and provided informational brochures designed to optimize the patients’ understanding of their pathology and perioperative course. Non–English language interpreters were readily available, and all the documents were available in English and Spanish. The patients also met with the anesthesia providers to discuss multimodal analgesia (gabapentin, regional anesthesia, oral narcotics) and postoperative pain control expectations. The physical therapist (PT) and occupational therapist (OT) provided an initial assessment of the patients’ functionality and discussed their postoperative mobility expectations (prior level of function, Boston University AM-PAC [activity measure for postacute care] basic mobility questionnaire, patient goals). A social worker or case manager screened the patients for any unanticipated discharge needs (durable medical equipment, Americans with Disabilities act compliance of home, in-home nursing needs) or barriers to discharge (family support, insurance needs). If time allowed, the patients were also offered peer counseling through the Hanger Prosthetic Clinic (Fresno, CA), the local prosthetic company. The decision to perform below-the-knee vs above-the-knee amputation was determined by the vascular examination findings and tissue viability. If the patient had evidence of adequate ileofemoral and profunda flow on the clinical examination and/or imaging study with adequate tissue to allow for coverage, a below-the-knee amputation was routinely performed. If significant inflow disease would preclude healing, for the appropriately selected patient with adequate tissue viability, revascularization was often performed to salvage a below-the-knee amputation.

Intraoperatively, all amputations were performed in the standard fashion. In addition to general anesthesia, locoregional anesthesia was used (ie, nerve block or epidural), unless contraindicated. The patients’ limbs were dressed with Kerlix bandages (Cardinal Health, Dublin, OH) and elastic wraps and were fit with a rigid removable limb protector on postoperative day (POD) 0. On POD 1, the Foley catheter, if placed, was removed, and the PT and OT met with the patient to begin therapeutic exercises. Referrals for postdischarge placement were also initiated by the social worker on POD 1. On POD 2, the surgical team evaluated the wound and cleared the patient for discharge if pain was controlled and the patient had no other inpatient hospital needs. If the patient was not ready for discharge on POD 2, the wound was reassessed and therapy continued until the patient had met the criteria for discharge. On discharge, the surgical team engaged the designated amputation coordinator, whose role was to ensure compliance with all follow-up appointments and who was available for patient outreach. At 3 weeks after the amputation, the surgical team examined the patient in the outpatient setting. At that time, the staples were removed, we provided a prescription for a stump shrinker and prosthesis, and referred the patient to the PT and OT. The patient then met with the prosthetist at approximately postoperative week 5 for the initial molding of the limb, with anticipation of receipt of the prosthesis by postoperative week 8. Follow-up with the surgeon occurred again at 3 months to ensure complete healing and no issues with ambulation.

The outcomes of our prospective patients were compared with those of historic control patients treated before the initiation of LEAP (January 2016 to December 2018). In the retrospective and prospective cohorts both, the demographic factors, operative details, interval until meeting with the PT and social worker, postoperative hospital LOS, discharge disposition, and postoperative outcomes (including morbidity and mortality, 30-day readmission, time to receipt of prosthesis, and time to ambulation) were collected. Prosthetic data were available through a database provided by the Hanger Prosthetic Clinic. If patients were not in the database, they were contacted to provide information on receipt of their prosthesis. The primary outcome of interest was the postoperative hospital LOS. The secondary outcomes of interest were unplanned readmissions, time to receipt of the prosthesis, and time to ambulation. The groups were compared using the Mann-Whitney *U* tests for continuous data and the Fisher exact test for categorical data. Statistical significance was defined as *P* < .05. Statistical analysis was performed using the SPSS, version 24.0 (IBM Corp, Armonk, NY).

## Results

A total of 141 patients were included in the present study with 130 in the retrospective control arm and 11 in the prospectively enrolled LEAP arm. All the patients were functionally independent before their hospitalization and all had had a diagnosis of PAD. The average age was 57 ± 13 years, and 73% were men. The predominant ethnicity was Hispanic (56%). The presence of multiple comorbidities was common, with diabetes mellitus (91%) and hypertension (89%) predominating, and >50% with a history of a minor lower extremity amputation. A comparative analysis between the retrospective and prospective cohorts was performed with no significant differences found in any demographic category (Table I).

**Table I:** Comparison analysis of retrospective and prospective cohorts

Variable	Total (N = 141)	Retrospective (n = 130)	LEAP (n = 11)	*P* value
Gender				1.00
Male	103 (73)	95 (73)	8 (73)	
Female	38 (27)	35 (27)	3 (27)	
Age, years	57 ± 13	57 ± 13	59 ± 14	.43
BMI, kg/m^2^	29 ± 8	29 ± 8	29 ± 8	.63
Race/ethnicity				.62
White	47 (33)	41 (32)	6 (54)	
Black	9 (6)	9 (7)	0 (0)	
Hispanic	79 (56)	74 (57)	5 (46)	
Asian	3 (2)	3 (2)	0 (0)	
Other	3 (2)	3 (2)	0 (0)	
Comorbidities				
Coronary artery disease	43 (30)	39 (30)	4 (36)	.74
Hypertension	125 (89)	114 (88)	11 (100)	.61
Diabetes mellitus	128 (91)	118 (91)	10 (91)	1.00
Smoker (former or current)	66 (47)	63 (48)	3 (27)	.22
End-stage renal disease	24 (17)	22 (17)	2 (18)	.92
Prior minor amputation	83 (59)	74 (57)	9 (82)	.13
Prior vascular intervention	29 (21)	26 (20)	3 (27)	.70
Primary amputation indication				.62
Infection	98 (70)	91 (70)	7 (64)	
Ischemia	25 (18)	22 (17)	3 (27)	
Both	18 (12)	17 (13)	1 (9)	
Preoperative ambulatory status				.60
Independent	100 (71)	93 (72)	7 (64)	
With assistance	20 (14)	19 (15)	1 (9)	
Wheelchair/walker but transfers	21 (15)	18 (14)	3 (27)	

*BMI,* Body mass index; *LEAP,* lower extremity amputation protocol.

Data presented as number (%) or mean ± standard deviation.

A below-the-knee amputation had been performed in 94% of the retrospective cohort and 100% of the prospective cohort. One patient in the prospective cohort had required an above-the-knee amputation 1 year after the index procedure because of progression of ischemia. A guillotine amputation had initially been performed for 43% of all patients, with no differences between the retrospective and prospective cohorts (*P* = 0.52). Formalization will typically occur within 48 to 72 hours of the initial amputation. The LEAP patients had had a significantly higher rate of the use of regional anesthesia than had the control group (100% vs 34%; *P* < .001) and had seen the PT and/or OT and the social worker sooner (2 days before surgery vs POD 0; *P* = .006; and 2 days before surgery vs POD 1; *P* = 0.001, respectively). Furthermore, the LEAP patients had been discharged an average of 3 days sooner than had the retrospective controls (6 days vs 3 days; *P* < .001) and had more often been discharged to an acute rehabilitation hospital (100% vs 27%; *P* < .001). No significant difference was found in the preoperative hospital LOS between the retrospective and LEAP cohorts (6 ± 6 days vs 7 ± 4 days; *P* = .53).

**Fig 2:**
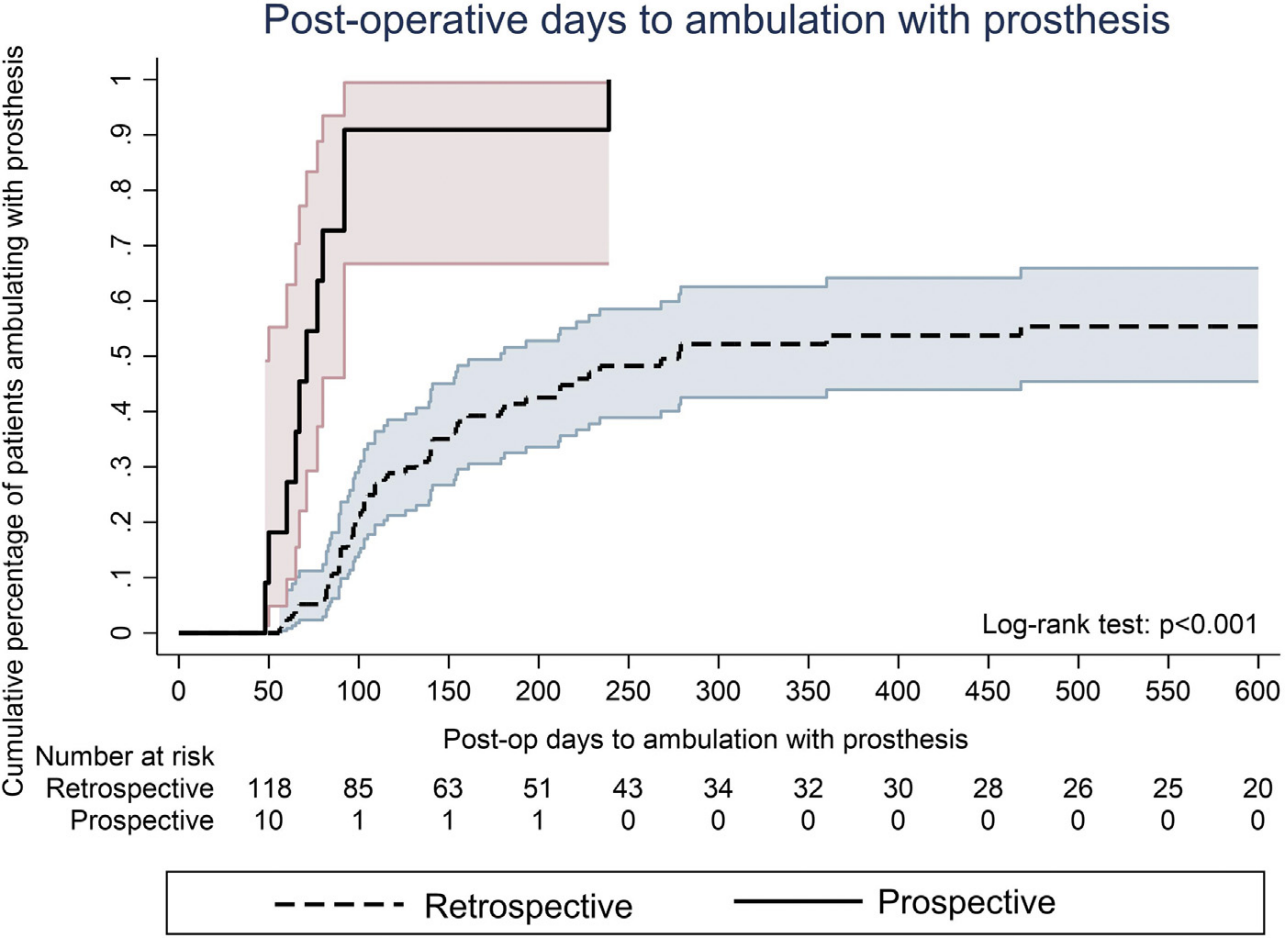
Graph showing postoperative (*Post-op*) days to ambulation with prosthesis stratified by cohort.

The mean follow-up was 351 ± 338 days (LEAP, 250 ± 171; vs retrospective, 360 ± 348 days; *P* = .62). After discharge, 100% of the LEAP patients had received their prosthesis compared with only 45% of the retrospective control patients (*P* < .001; Table II). Of the patients who had received their prosthesis, the LEAP patients had received their prosthesis, on average, 2 months sooner than had the retrospective cohort (81 ± 39 days vs 137 ± 97 days, respectively; *P* = .002) and had ambulated with their prosthesis sooner than had the patients in the control group (86 ± 53 days vs 146 ± 104 days, respectively; *P* = .002). The Kaplan-Meier estimates of the postoperative rate of ambulation with the prosthesis for the retrospective vs LEAP patients are presented in Fig 2. No significant differences were found in the 30-day readmissions, rate of surgical site infection, major adverse cardiac events, or mortality between the two cohorts.

**Table II:** Outcomes analysis stratified by cohort

Outcome	Total (N = 141)	Retrospective (n = 130)	LEAP (n = 11)	*P* value
Preoperative LOS, days	6 ± 6	6 ± 6	7 ± 4	.53
Guillotine amputation	64 (43)	60 (46)	4 (36)	.75
Formalized amputation				1.00
Above the knee	8 (6)	8 (6)	0 (0)	
Below the knee	133 (94)	122 (94)	11 (100)	
Regional block used	55 (39)	44 (34)	11 (100)	**< .001**
Perioperative gabapentin	57 (40)	52 (50)	5 (46)	.76
Foley catheter removed, days	0 ± 2	0 ± 2	0 ± 0	.72
Follow-up, days	351 ± 338	360 ± 348	250 ± 171	.62
Postoperative time to PT, days	0 ± 4	0 ± 4	−2 ± 4	**.006**
Postoperative time to social worker, days	1 ± 4	1 ± 4	−2 ± 3	**.001**
Total LOS, days	12 ± 9	12 ± 9	9 ± 4	.28
Guillotine	12 ± 5	12 ± 5	10 ± 4	
Single stage	13 ± 11	13 ± 11	9 ± 5	
Postoperative LOS, days	6 ± 5	6 ± 5	3 ± 2	**< .001**
Discharge disposition				**< .001**
Skilled nursing facility	44 (31)	44 (34)	0 (0)	
Rehabilitation facility	46 (33)	35 (27)	11 (100)	
Home	51 (36)	51 (39)	0 (0)	
Received prosthesis	69 (49)	58 (45)	11 (100)	**< .001**
Time to prosthesis, days	128 ± 93	137 ± 97	81 ± 39	**.002**
Ambulated	67 (48)	56 (43)	11 (100)	**< .001**
Time to ambulation, days	136 ± 100	146 ± 104	86 ± 53	**.002**
Readmission within 30 days	29 (21)	26 (20)	3 (27)	.70
Surgical site infection	19 (14)	19 (15)	0 (0)	.36
Major adverse cardiac event	3 (2)	3 (2)	0 (0)	1.00
Overall mortality	9 (6)	9 (7)	0 (0)	1.00
Mortality ≤1 year of major amputation	4 (3)	4 (3)	0 (0)	1.00

*LEAP,* Lower extremity amputation protocol; *LOS,* length of stay; *PT,* physical therapy.

Data presented as mean ± standard deviation or number (%).

Boldface *P* values represent statistical significance.

## Discussion

We designed LEAP to close previously identified gaps in the treatment of vascular patients requiring a lower extremity amputation by using the principles of ERAS with the goal of decreasing the postoperative hospital LOS and expediting the time to functional ambulation with a prosthesis. We prospectively enrolled 11 consecutive patients who were undergoing a major amputation and found that, compared with their retrospective cohort, the LEAP patients had had a significantly decreased postoperative hospital LOS, higher rate of ambulation with a prosthesis, and a faster time to ambulation. Additionally, no differences in surgical morbidity or unplanned readmissions were found between the two cohorts, demonstrating that the use of LEAP is safe and leads to improved outcomes for this patient population.

ERAS programs have been widely used across surgical subspecialties and have clearly demonstrated improved outcomes. However, prior studies have suggested that these protocols must be tailored to the patient population and type of surgery to optimize success.^15^^,^^17^^,^^18^ Historically, the STAMP (special team for amputation, mobility, prosthetic) program through the U.S. Department of Veterans Affairs demonstrated promising results using a multidisciplinary approach for amputees in the 1980s.^19^ However, few contemporary data in vascular surgery are available regarding postoperative protocols, with a few studies of ERAS protocols for aortic and lower extremity bypass procedures, all with promising results.^20^, ^21^, ^22^ Furthermore, these protocols require a collaborative effort between healthcare professionals within a hospital system, as well as patient participation, all of which can be barriers to protocol implementation. Studies have shown that building a multidisciplinary team with strong leadership, preoperative patient education, and postdischarge continuity of care will all contribute to the success of any ERAS program.^17^^,^^18^^,^^23^ LEAP uses comprehensive medical (surgery and anesthesia) and ancillary (social work, physical therapy, occupational therapy, and prosthetic) teams, in addition to hospital nursing staff, who work collaboratively to ensure the protocol is followed.

The initial goal of LEAP was to decrease the overall hospital LOS to minimize the burden for both the patients and the healthcare system and to address the negative outcomes related to a prolonged hospital LOS. A common cause of an unnecessarily prolonged hospital LOS is a lack of communication between the medical providers and ancillary services, which leads to delays in treatment and, ultimately, prolonged hospitalization. Using LEAP preoperatively, all disciplines will be informed of the patient and can enact their roles with efficiency. This was evident by the 100% use of regional anesthesia and the PT, OT, and social worker consultations occurring place preoperatively and a postoperative hospital LOS reduced by 3 days compared with the control group.

Previous studies have suggested that placement at an acute rehabilitation hospital after surgery will lead to a shorter time to ambulation and decreased overall disability.^12^^,^^24^ All LEAP patients were discharged to an acute rehabilitation hospital, irrespective of their insurance status. Consulting with the social worker early facilitated communication with the rehabilitation facilities, allowed timely initiation of the required paperwork, and enabled uninsured patients to obtain at least temporary insurance status. Additionally, the use of a dedicated amputation coordinator allowed for continuity of care and compliance with follow-up and gave the patients access to a “hotline” should they have any questions or concerns regarding their postoperative course. The role of a coordinator or navigator for patient care has been well established among many medical specialties with clear patient benefits, and one can only surmise that this benefit would certainly extend to many facets of vascular surgery, including the amputee population.^25^, ^26^, ^27^

The ultimate goal of LEAP is to afford vascular patients undergoing major lower extremity amputations every opportunity to receive a prosthesis and successfully ambulate independently. By allowing the prosthetist to visit and deliver the limb protector during the patients’ hospital stay, a relationship was established with the patient, providing another avenue for continuity of care and additional support. The ability to ensure receipt of a prosthesis and independently ambulate nearly 2 months sooner will, not only offload a huge personal burden to the patient, but also decrease overall healthcare usage. Independent ambulation allows for patients, not only to return home as functional members of their family unit, but also to decrease work loss days resulting from the disability.

One half of the patients in the study were Hispanic, a known underserved ethnicity that has been demonstrated to have an elevated risk of diabetes and higher odds of amputation when hospitalized with PAD compared with non–Hispanic races.^28^, ^29^, ^30^ Chen et al^28^ noted in a cross-sectional analysis of >300,000 inpatient hospitalizations for PAD that the average age of the Hispanic population was 57 years and had presented with advanced-stage disease, consistent with our findings. This suggests that perhaps the Hispanic population might be affected at a younger age, in addition to being underdiagnosed and undertreated.^28^ Achieving improved outcomes for underserved populations emphasizes the importance of implementing a standardized postamputation protocol across all patient populations to help decrease healthcare disparities. Additionally, our findings have highlighted the need for further research efforts in screening and early treatment in this high-risk population.

The present study had several limitations. Our prospective pilot trial was subject to the selection bias inherent to most clinical trials (availability of staff for the consent process, patient agreement). The COVID-19 (coronavirus disease 2019) pandemic that began in March 2020 led to decreased hospital resources, conversion of the acute rehabilitation hospitals to overflow patient care units, and limited access to outpatient prosthetic services. Enrollment in clinical trials at UCSF-Fresno was also halted during this time, a part of the reason for the enrollment of only 11 patients. However, with the promising results, multidisciplinary enthusiasm, and demonstrated safety of the protocol, the hospital system has decided to use LEAP across all three of its community medical center hospitals within the metropolitan area of Fresno, California, as the standard of care for all amputees. Thus, future higher powered outcomes data on the protocol can be expected. Additionally, the nonrandomized nature of this prospective trial could have induced a certain level of selection bias, which was unavoidable. Only retrospective patients who were functionally ambulatory before their amputation were included in the retrospective cohort in attempt to achieve similarity between the two groups. On univariate analysis, no statistically significant differences were found in the demographics of the two cohorts. Although all prospective patients had completed quality of life questionnaires postoperatively, this had not been previously mandated; therefore, no comparison with the retrospective cohort was possible. Finally, the retrospectively collected data were limited by the information available from the electronic health records.

## Conclusions

The results from our pilot study have demonstrated that LEAP can significantly decrease the postoperative LOS and expedite the time to independent ambulation with a prosthesis for vascular patients undergoing major lower extremity amputation. These findings suggest a powerful ability to bridge the healthcare gap for this high-risk, underserved, and ethnically diverse population using a disease-specific standardized protocol.
